# Impact of surgeon volume, experience, and training on outcomes after arthroscopic rotator cuff repair: a nationwide analysis of 1489 surgeons

**DOI:** 10.1016/j.jseint.2024.04.004

**Published:** 2024-04-27

**Authors:** Ryan Sadjadi, Cameron Nosrat, Favian Su, Drew A. Lansdown, Brian T. Feeley, C. Benjamin Ma, Alan L. Zhang

**Affiliations:** Department of Orthopaedic Surgery, University of California, San Francisco, San Francisco, CA, USA

**Keywords:** Arthroscopic rotator cuff repair, Surgeon-specific factors, Reoperations, Hospitalizations, Revision surgery, Emergency department visits

## Abstract

**Background:**

Given the complexity of arthroscopic rotator cuff repair (ARCR) and increasing prevalence, there is a need for comprehensive, large-scale studies that investigate potential correlations between surgeon-specific factors and postoperative outcomes after ARCR. This study examines how surgeon-specific factors including case volume, career length, fellowship training, practice setting, and regional practice impact two-year reoperation rates, conversion to total shoulder arthroplasty (anatomic or reverse), and 90-day post-ARCR hospitalization.

**Methods:**

The PearlDiver Mariner database was used to collect surgeon-specific variables and query patients who underwent ARCR from 2015 to 2018. Patient outcomes were tracked for two years, including reoperations, hospitalizations, and International Classification of Diseases, Tenth Revision codes for revision rotator cuff repair (RCR) laterality. Hospitalizations were defined as any emergency department (ED) visit or hospital readmission within 90 days after primary ARCR. Surgeon-specific factors including surgeon case volume, career length, fellowship training, practice setting, and regional practice were analyzed in relation to postoperative outcomes using both univariate and multivariate logistic regression.

**Results:**

94,150 patients underwent ARCR by 1489 surgeons. On multivariate analysis, high-volume surgeons demonstrated a higher risk for two-year total reoperation (odds ratio [OR] = 1.06, 95% confidence interval [CI]: 1.01-1.12, *P* = .03) and revision RCR (OR = 1.06, 95% CI: 1.01-1.12, *P* = .02) compared to low-volume surgeons. Early-career surgeons showed higher rates of 90-day ED visits (mid-career surgeons: OR = 0.78, 95% CI: 0.73-0.83, *P* < .001; late-career surgeons: OR = 0.73, 95% CI: 0.68-0.78, *P* < .001) and hospital readmission (mid-career surgeons: OR = 0.74, 95% CI: 0.63-0.87, *P* < .001; late-career surgeons: OR = 0.73, 95% CI: 0.61-0.88, *P* = .006) compared to mid- and late-career surgeons. Sports medicine and/or shoulder and elbow fellowship-trained surgeons demonstrated lower two-year reoperation risk (OR = 0.95, CI: 0.91-0.99, *P* = .04) and fewer 90-day ED visits (OR = 0.93, 95% CI = 0.88-0.98, *P* = .002). Academic surgeons experienced higher readmission rates compared to community surgeons (OR = 1.16, 95% CI = 1.01-1.34, *P* = .03). Surgeons practicing in the Northeast demonstrated lower two-year reoperation (OR = 0.88, 95% CI: 0.83-0.93, *P* < .001) and revision (OR = 0.88, 95% CI: 0.83-0.94, *P* < .001) RCR risk compared to surgeons in the Southern United States.

**Conclusion:**

High-volume surgeons exhibit higher two-year reoperation rates after ARCR compared to low-volume surgeons. Early-career surgeons demonstrate increased hospitalizations. Sports medicine or shoulder and elbow surgery fellowships correlate with reduced two-year reoperation rates and 90-day ED visits.

Arthroscopic rotator cuff repair (ARCR) is a commonly performed procedure with an estimated incidence of 165 repairs per 100,000 person-years.^2^^,^^3^,^30^ A large proportion of these procedures experience complications, such as retears that necessitate further surgery.^17^ Specifically, a systematic review and meta-analysis of 31 studies investigating retear rates following rotator cuff repair (RCR) found the 12-24-month postoperative retear rate to be 21%.^18^ Previous research has investigated various patient-related factors that might influence outcomes and revisions. These factors include age, body mass index, diabetes, tobacco use, and dyslipidemia.^1^^,^^4^^,^^6^^,^^10^^,^^12^^,^^14^^,^^20^^,^^21^^,^^26^^,^^28^ Additionally, tear characteristics, such as size, retraction, muscle atrophy, fatty infiltration, and chronicity, have also been shown to influence revision rates.^7^, ^8^, ^9^^,^^11^^,^^31^ However, there is limited evaluation on how surgeon-specific factors might impact the outcomes of ARCRs.

There has been increasing interest in understanding the role of surgeon experience and case volume on postoperative outcomes. In orthopedic surgery, outcomes are often superior when procedures, such as hip, knee, and shoulder arthroplasties, are undertaken by surgeons with extensive experience and high-case volumes.^13^^,^^19^^,^^24^ However, a recent study examining the relationship between surgeon case volume and experience and 1-year patient-reported outcome measures found no such association following primary ARCR.^25^ The study suggested that a possible convergence in surgical skill among surgeons in the study might explain these findings.^25^ Given the procedure's inherent complexity and increasing prevalence, there is a need for comprehensive, large-scale studies that investigate potential correlations between surgeon-specific factors and outcomes after ARCR.

Therefore, the purpose of this study was to use a large administrative database to evaluate the impact of surgeon factors, including case volume, career duration, fellowship training, practice type, and region of practice, on the risk of two-year revisions and 90-day hospitalizations following primary ARCR. Hospitalizations were defined as any emergency department (ED) visit or hospital readmission within 90 days after primary ARCR. We hypothesize that surgeons with a higher case volume and longer career duration will have a lower risk of reoperations and 90-day hospitalizations, whereas fellowship training, practice setting, and region of practice will have no effect.

## Methods

### Data extraction

Data for this study were extracted from the Pearldiver Mariner database (PearlDiver Technologies, Colorado Springs, CO, USA). This database houses clinical and demographic data for over 157 million US patients, encompassing a range of coverage and payment modalities including private insurance, Medicare, Medicaid, and cash payments. Current Procedural Terminology (CPT) codes and International Classification of Diseases, Tenth Revision (ICD-10) diagnoses were utilized to source data. All details obtained from PearlDiver are anonymous and therefore were exempt from institutional review board approval.

### Study cohort

Between 2015 and 2018, individuals who had undergone ARCRs were identified by referring to relevant CPT and ICD-10 diagnostic codes (see Supplementary Table S1 for details). Surgeons handling these procedures were queried within the database through their names and associated National Provider Identifier credentials. The total number of cases for each surgeon was totaled, and only those who performed more than 10 arthroscopic rotator cuff surgeries within the specified study period were included. Surgeons without National Provider Identifier credentials were excluded. Subsequently, we collected supplementary surgeon information from publicly available online repositories, which included gender, race/ethnicity, practice region (divided into Northeast, Midwest, South, and West), post-residency practice tenure, educational qualifications (MD or DO), specifics of their advanced training (sports medicine, shoulder and elbow, arthroplasty, or trauma), and their practice setting (academic or community-based).

### Surgeon-specific variables

Surgeon-specific variables including case volume, career duration, fellowship training, practice region, and practice type were assessed from PearlDiver outputs. Case volume was stratified into tertiles based on the number of ARCRs performed between 2015 and 2018. One-third of the surgeon cohort was categorized as “low volume” (<113 cases), another third as “medium volume” (113-200 cases), and the remaining under “high volume” (>200 cases). Surgeon career duration was also stratified into tertiles, with those who had completed residency between 2009 and 2018 (<10 years in practice) considered “early-career”, 1999-2008 (10-20 years in practice) considered “mid-career”, and before 1999 (>20 years in practice) considered “late-career”.

### Study outcomes

Over a two-year period following the initial RCR, patients were tracked for subsequent reoperations on the ipsilateral shoulder. These included revision RCR, conversion to total shoulder arthroplasty (including anatomic or reverse) (TSA), and other arthroscopic and open shoulder surgery procedures such as biceps tenodesis (Supplementary Table S2). Using the relevant CPT and ICD-10 codes, the laterality of the reoperations was matched to the index surgery. Additionally, 90-day hospitalizations, defined in this investigation as hospital readmissions and emergency room encounters within 90 days after primary ARCR, were tracked. The rates of two-year reoperation and 90-day hospital admissions were correlated with the previously mentioned surgeon-specific factors.

### Statistical analysis

Surgeon demographic data are expressed as means with standard deviations.

Univariate statistical analyses were employed to ascertain associations between surgeon-specific variables (surgery volume, career duration, fellowship training, practice type, and region of practice) and total reoperations, revision rotator cuff arthroscopy, conversion to TSA, and 90-day hospitalizations, ED visits, and readmissions. Chi-squared tests were used to analyze categorical variables. T-tests were conducted for continuous variables with two groups, and one-way analysis of variance was employed for continuous variables with more than two groups. A multivariate logistic regression analysis was performed to isolate surgeon-specific factors that independently correlated with specified outcome metrics. The analysis adjusted for patient factors including age, sex, comorbidities associated with diabetes and tobacco use, and the Charlson comorbidity index. Statistical significance was established at a *P* value of ≤.05. Data analyses were carried out using JMP Pro (version 16; SAS Institute, Cary, NC, USA) and the R statistical package (version 2022.02.3, 2022; Boston, MA, USA).

## Results

In this cohort, there were 94,150 patients who underwent ARCR between October 2015 and December 2018 by 1489 surgeons. The two-year total reoperation rate for patients was 9.3% (8781 patients), with 6.9% representing revision rotator cuff arthroscopy procedures (6517 patients) and 0.4% representing conversions to TSA (406 patients). The overall 90-day hospitalization rate was 7.4% (7005 patients), with 6.9% representing ED admissions (6469 patients) and 0.9% representing hospital readmissions (890 patients).

### Surgeon demographics

Of 1489 surgeons, 97.2% (n = 1447) identified as men and 2.8% (n = 42) as women (Table I). Additionally, 86.0% of surgeons (n = 1281) identified as white and 14.0% (n = 208) as non-white (Table I). Medical school distribution showed 91.7% (n = 1365) had an MD degree and 8.3% (n = 124) had a DO degree (Table I).

**Table I:** Demographic and clinical distribution of surgeons and patients for arthroscopic rotator cuff repair categorized by surgeon gender, race, and degree type.

Surgeon demographic	Gender		Race		Degree type	
	Men	Women	White	Non-White	MD	DO
Number of surgeons (%)	1447 (97.2)	42 (2.8)	1281 (86.0)	208 (14.0)	1365 (91.7)	124 (8.30)
Number of patients (%)	90,883 (96.5)	3267 (3.5)	79,599 (84.5)	14,551 (15.5)	86,651 (92.0)	7499 (8.0)
Mean case volume	218.0	297.6	215.4	250.2	220.9	213.0
Patient age∗	60.5 ± 9.4	60.7 ± 9.2	60.5 ± 9.4	60.4 ± 9.4	60.5 ± 9.4	60.3 ± 9.4
Patient sex, % female	47.1	50.3	46.9	48.8	47.1	48.2

∗ Data presented as mean ± standard deviation.

### Surgeon volume

**Figure 1:**
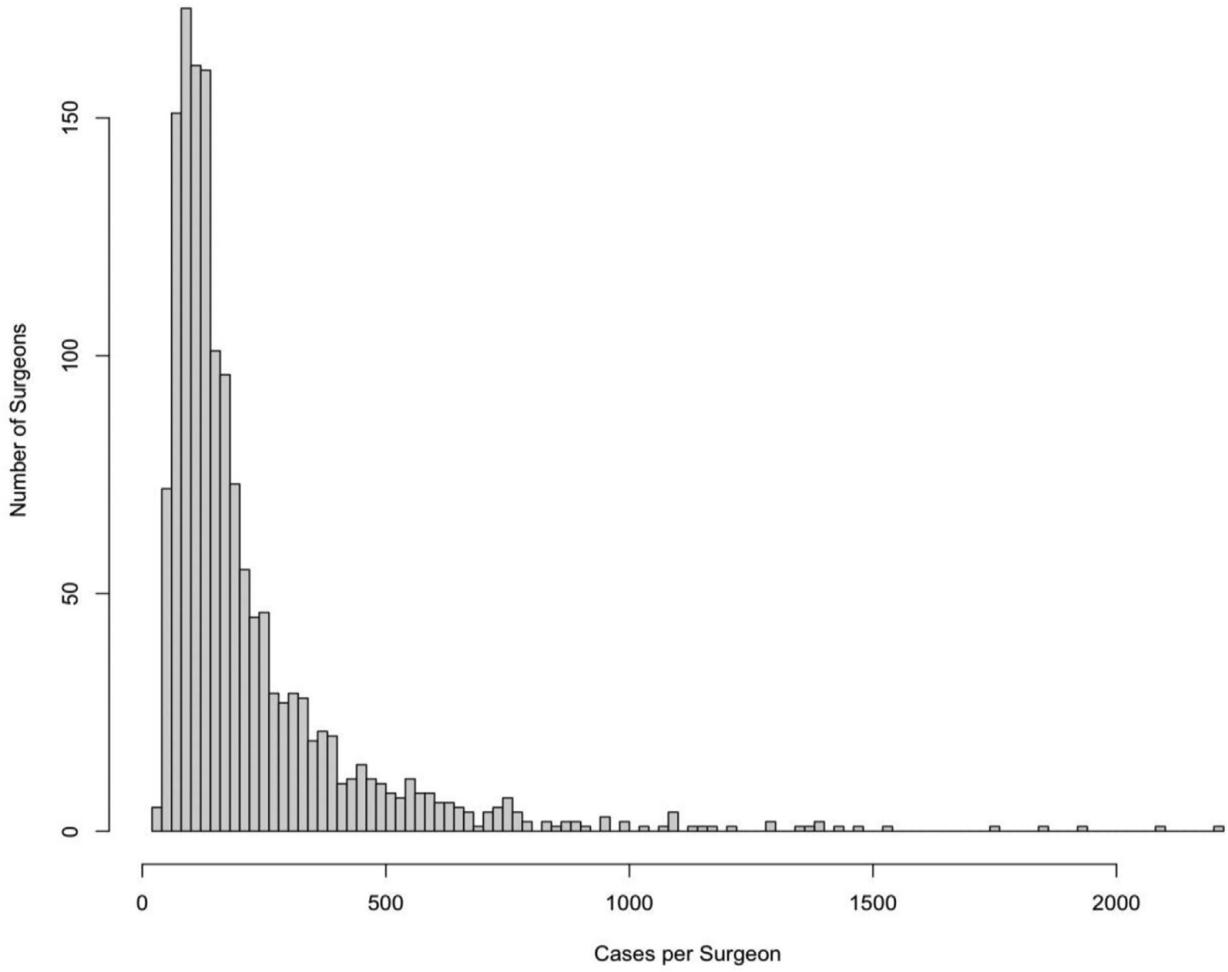
Arthroscopic rotator cuff repair (ARCR) case volume distribution. Number of ARCR procedures during the study period for each surgeon in the database (total 1489 surgeons).

We identified 495 low-volume surgeons, 497 medium-volume surgeons, and 497 high-volume surgeons (Table II). The distribution of ARCR case volume across the entire cohort is depicted in Figure 1. The mean case volume of low-, medium-, and high-volume surgeons was 81.7 ± 18.2, 148.8 ± 24.7, and 429.7 ± 283.8, respectively (*P* < .001). Additionally, there was a significant difference in patient age (*P* < .001) among the groups. On univariate analysis, low- and medium-volume surgeons were found to have significantly higher rates of two-year conversion to TSA compared to high-volume surgeons (*P* < .001; Table II). Additionally, medium-volume surgeons had significantly higher rates of 90-day hospital readmission compared to low- and high-volume surgeons (*P* = .004; Table II).

**Table II:** Patient distribution stratified by surgeon volume.

	Low volume	Medium volume	High volume	*P* value
Number of surgeons (%)	495 (33.2%)	497 (33.4%)	497 (33.4%)	
Number of patients (%)	30,465 (32.4%)	32,289 (34.3%)	31,396 (33.3%)	
Mean case volume	81.7	148.8	429.7	
Patient age∗	60.7 ± 9.4	60.4 ± 9.4	60.4 ± 9.4	**<.001**
Patient sex, % female	46.8	46.6	47.4	.11
Reoperation within 2 years†	2806 (9.2%)	3071 (9.5%)	2921 (9.3%)	.41
Revision arthroscopic rotator cuff repair within 2 years†	2084 (6.8%)	2279 (7.1%)	2162 (6.9%)	.52
Conversion to TSA within 2 years†	137 (0.4%)	145 (0.4%)	46 (0.1%)	**<.001**
90-day hospitalization†	2079 (6.8%)	2335 (7.2%)	2271 (7.2%)	.07
90-day ED visit†	1944 (6.4%)	2125 (6.6%)	2133 (6.8%)	.12
90-day hospital readmission†	233 (0.8%)	323 (1.0%)	261 (0.8%)	**.004**

*TSA*, total shoulder arthroplasty; *ED*, emergency department.

Bold values indicate statistical significance.

∗ Data presented as mean ± standard deviation.

† Data presented as number (%) of patients undergoing procedure from index cohort of patients who underwent arthroscopic rotator cuff repair.

### Career duration

**Figure 2:**
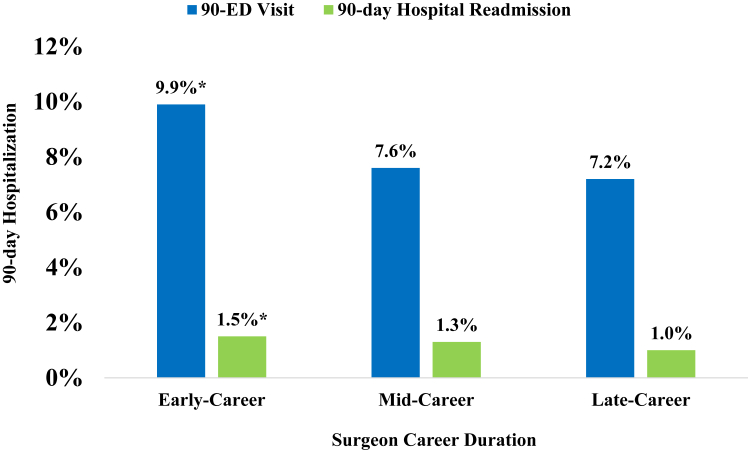
90-day hospitalization rates by surgeon career duration stratification. Rates of 90-day emergency department visits and 90-day hospital readmission within 2 years of index arthroscopic rotator cuff repair stratified by early-, mid-, and late-career surgeons. ∗Indicates statistically significant difference.

We identified 341 early-career surgeons, 594 mid-career surgeons, and 554 late-career surgeons (Table III). There were significant differences in patient demographics, including patient age (*P* < .001) and sex (*P* < .001), between surgeons of each career duration (Table III). There were significant differences in rates of 90-day hospitalization (*P* < .001), ED visits (*P* < .001), and hospital readmission (*P* < .001) when comparing surgeons of different career durations with early-career surgeons having the highest risk for hospitalizations after surgery (Table III, Fig. 2). In the study cohort, only 20.1% of ARCRs were performed by early-career surgeons.

**Table III:** Patient distribution stratified by surgeon career duration.

	Early-career	Mid-career	Late-career	*P* value
Number of surgeons (%)	341 (22.9%)	594 (39.9%)	554 (37.2%)	
Number of patients (%)	19,139 (20.3%)	40,321 (42.8%)	34,690 (36.8%)	
Mean case volume	193.1	237.5	218.4	
Patient age∗	59.8 ± 9.4	60.2 ± 9.5	60.8 ± 9.4	**<.001**
Patient sex, % female	47.2	46.7	45.3	**<.001**
Reoperation within 2 years†	1753 (9.2%)	3795 (9.4%)	3264 (9.4%)	.57
Revision arthroscopic rotator cuff repair within 2 years†	1320 (6.9%)	2807 (7.0%)	2414 (7.0%)	.95
Conversion to TSA within 2 years†	86 (0.4%)	189 (0.5%)	157 (0.5%)	.93
90-day hospitalization†	2063 (10.8%)	3425 (8.5%)	2710 (7.8%)	**<.001**
90-day ED visit†	1896 (9.9%)	3084 (7.6%)	2482 (7.2%)	**<.001**
90-day hospital readmission†	289 (1.5%)	526 (1.3%)	352 (1.0%)	**<.001**

*TSA*, total shoulder arthroplasty; *ED*, emergency department.

Bold indicates statistical significance with *P* ≤ .05.

∗ Data presented as mean ± standard deviation.

† Data presented as number (%) of patients undergoing procedure from index cohort of patients who underwent arthroscopic rotator cuff repair.

### Fellowship training

Of the 1489 surgeons included in our sample, 668 (44.9%) had fellowship training in sports medicine and/or shoulder and elbow while 821 (55.1%) did not (Table IV). There were significant differences in patient demographics, including age (*P* < .001) and sex (*P* = .01) between surgeons of each fellowship training classification. Additionally, surgeons without fellowship training in sports medicine and/or shoulder and elbow surgery had a significantly higher rate of two-year conversion to TSA (0.5%, *P* = .05) and 90-day ED visits (6.8%, *P* = .04) compared to those with fellowship training in sports medicine and/or shoulder and elbow surgery (Table IV).

**Table IV:** Patient distribution stratified by surgeon fellowship training.

	Sports medicine and/or shoulder and elbow	Other fellowship training	*P* value
Number of surgeons (%)	668 (44.9%)	821 (55.1%)	
Number of patients (%)	42,028 (44.6%)	52,122 (55.4%)	
Mean case volume	220.2	220.3	
Patient age∗	60.2 ± 9.4	60.7 ± 9.4	**<.001**
Patient sex, % female	46.4	47.2	**.01**
Reoperation within 2 years†	3863 (9.2%)	4920 (9.4%)	.19
Revision arthroscopic rotator cuff repair within 2 years†	2862 (6.8%)	3656 (7.0%)	.22
Conversion to TSA within 2 years†	172 (0.4%)	258 (0.5%)	**.05**
90-day hospitalization†	2966 (7.1%)	3803 (7.3%)	.15
90-day ED visit†	2723 (6.5%)	3552 (6.8%)	**.04**
90-day hospital readmission†	387 (0.9%)	443 (0.8%)	.24

*TSA*, total shoulder arthroplasty; *ED*, emergency department.

Bold indicates statistical significance with *P* ≤ .05.

∗ Data presented as mean ± standard deviation.

† Data presented as number (%) of patients undergoing procedure from index cohort of patients who underwent arthroscopic rotator cuff repair.

### Practice type

568 (38.1%) of the surgeons in this cohort were determined to have an academic practice while 921 (61.9%) were determined to have a community-based practice (Table V). There were no significant differences in sex, reoperation, and revision ARCR within 2 years (Table V). There was a significant difference in patient age (*P* < .001) between academic and community-based surgeons. Furthermore, the rate of two-year conversion to TSA was significantly lower for academic surgeons (0.4%, *P* = .04) compared to community-based surgeons while 90-day hospitalization (6.9%, *P* = .007), ED visit (6.4%, *P* = .04), and hospital readmission (0.8%, *P* = .04) rates were significantly lower for community-based surgeons compared to academic surgeons (Table V). In the study cohort, community-based surgeons performed 58.8% of ARCRs.

**Table V:** Patient distribution stratified by surgeon practice type.

	Academic	Community	*P* value
Number of surgeons (%)	568 (38.1%)	921 (61.9%)	
Number of patients (%)	38,009 (40.3%)	56,240 (59.7%)	
Mean case volume	238.2	209.2	
Patient age∗	60.1 ± 9.3	60.8 ± 9.4	**<.001**
Patient sex, % female	46.6	47.2	.07
Reoperation within 2 years†	3470 (9.1%)	5311 (9.4%)	.10
Revision arthroscopic rotator cuff repair within 2 years†	2589 (6.8%)	3931 (7.0%)	.29
Conversion to TSA within 2 years†	175 (0.4%)	255 (0.5%)	**.04**
90-day hospitalization†	2799 (7.4%)	3887 (6.9%)	**.007**
90-day ED visit†	2577 (6.8%)	3626 (6.4%)	**.04**
90-day hospital readmission†	358 (0.9%)	459 (0.8%)	**.04**

*TSA*, total shoulder arthroplasty; *ED*, emergency department.

Bold indicates statistical significance with *P* ≤ .05.

∗ Data presented as mean ± standard deviation.

† Data presented as number (%) of patients undergoing procedure from index cohort of patients who underwent arthroscopic rotator cuff repair.

### Region of practice

Based on regions within the United States, 395 (26.5%) practiced in the Midwest, 311 (20.9%) practiced in the Northeast, 593 (39.8%) practiced in the South, and 190 (12.8%) practiced in the West (Table VI). There were significant differences in patient demographics, including patient age (*P* < .001) and sex (*P* < .001), between surgeons of each region of practice in the United States. Additionally, there were significant differences in rates of two-year reoperation (*P* = .001), revision RCR (*P* < .001), conversion to TSA (*P* < .001), 90-day hospitalization (*P* < .001), and ED visits (*P* < .001) between groups (Table VI).

**Table VI:** Patient distribution stratified by surgeon region of practice.

	Midwest	Northeast	South	West	*P* value
Number of surgeons (%)	395 (26.5%)	311 (20.9%)	593 (39.8%)	190 (12.8%)	
Number of patients (%)	26,427 (28.1%)	19,691 (20.9%)	37,194 (39.5%)	10,838 (11.5%)	
Mean case volume	239.2	224.1	217.6	182.8	
Patient age∗	60.0 ± 9.3	59.8 ± 9.1	60.9 ± 9.5	61.7 ± 9.6	**<.001**
Patient sex, % female	45.4	46.7	48.1	47.6	**<.001**
Reoperation within 2 years†	2494 (9.4%)	1687 (8.6%)	3501 (9.4%)	1049 (9.7%)	**.001**
Revision arthroscopic rotator cuff repair within 2 years†	1839 (7.0%)	1235 (6.3%)	2612 (7.0%)	797 (7.4%)	**<.001**
Conversion to TSA within 2 years†	153 (0.6%)	84 (0.4%)	130 (0.3%)	45 (0.4%)	**<.001**
90-day hospitalization†	2001 (7.6%)	1336 (6.8%)	2531 (6.8%)	817 (7.5%)	**<.001**
90-day ED visit†	1878 (7.1%)	1225 (6.2%)	2341 (6.3%)	758 (7.0%)	**<.001**
90-day hospital readmission†	233 (0.9%)	178 (0.9%)	314 (0.8%)	92 (0.8%)	.88

*TSA*, total shoulder arthroplasty; *ED*, emergency department.

Bold indicates statistical significance with *P* ≤ .05.

∗ Data presented as mean ± standard deviation.

† Data presented as number (%) of patients undergoing procedure from index cohort of patients who underwent arthroscopic rotator cuff repair.

### Multivariate analysis

In the logistic regression analysis, high-volume surgeons demonstrated a higher risk of total reoperations (odds ratio [OR] = 1.06, 95% confidence interval [CI]: 1.01-1.12, *P* = .03) and revision RCR (OR = 1.06, 95% CI: 1.01-1.12, *P* = .02) as well as 90-day ED visits (OR = 1.06, 95% CI: 1.01-1.13, *P* = .05) compared to low-volume surgeons. Medium-volume surgeons displayed a higher propensity for 90-day hospital readmissions (OR = 1.36, 95% CI: 1.15-1.62, *P* < .001; Table VII). In addition, mid- and late-career surgeons demonstrated significantly lower 90-day hospital readmission (mid-career surgeons: OR = 0.74, 95% CI: 0.63-0.87, *P* < .001; late-career surgeons: OR = 0.73, 95% CI: 0.61-0.88, *P* = .006) and ED visit rates (mid-career surgeons: OR = 0.78, 95% CI: 0.73-0.83, *P* < .001; late-career surgeons: OR = 0.73, 95% CI: 0.68-0.78, *P* < .001) compared to early-career surgeons.

**Table VII:** Multivariate analysis.

Variable	Rotator cuff reoperation within 2 years		Rotator cuff revision within 2 years		Conversion to TSA within 2 years		90-day ED visits		90-day hospital readmissions	
	Parameter estimate (95% CI)∗	*P*	Parameter estimate (95% CI)∗	*P*	Parameter estimate (95% CI)∗	*P*	Parameter estimate (95% CI)∗	*P*	Parameter estimate (95% CI)∗	*P*
Surgical volume†										
Medium	1.03 [0.98, 1.09]	.26	1.04 [0.98, 1.09]	.21	0.89 [0.68, 1.17]	.42	1.04 [0.98, 1.11]	.23	1.36 [1.15, 1.62]	**<.001**
High	1.06 [1.01, 1.12]	**.03**	1.06 [1.01, 1.12]	**.02**	1.01 [0.77, 1.31]	.96	1.06 [1.01, 1.13]	**.05**	1.08 [0.90, 1.29]	.39
Career duration‡										
Mid	0.97 [0.92, 1.03]	.36	0.97 [0.92, 1.03]	.38	0.99 [0.75, 1.33]	.97	0.78 [0.73, 0.83]	**<.001**	0.74 [0.63, 0.87]	**<.001**
Late	1.01 [0.95, 1.07]	.81	1.01 [0.95, 1.08]	.69	0.88 [0.65 1.20]	.42	0.73 [0.68, 0.78]	**<.001**	0.73 [0.61, 0.88]	**.006**
Fellowship training type§										
Sports medicine and/or shoulder and elbow	0.95 [0.91, 0.99]	**.04**	0.96 [0.91, 1.00]	.06	0.94 [0.75, 1.18]	.59	0.93 [0.88, 0.98]	**.002**	1.06 [0.92, 1.22]	.37
Practice type‖										
Academic	1.01 [0.97, 1.06]	.66	1.02 [0.97, 1.06]	.49	0.81 [0.64, 1.02]	.08	1.05 [0.99, 1.11]	.07	1.16 [1.01, 1.34]	**.03**
Region¶										
Midwest	0.98 [0.93, 1.04]	.57	0.97 [0.92, 1.02]	.26	1.62 [1.25, 2.10]	**.002**	1.08 [1.01, 1.15]	**.01**	0.98 [0.82, 1.16]	.80
Northeast	0.88 [0.83, 0.93]	**<.001**	0.88 [0.83, 0.94]	**<.001**	1.02 [0.74, 1.40]	.89	0.96 [0.89, 1.04]	.31	1.07 [0.89, 1.29]	.48
West	1.06 [0.98, 1.14]	.10	1.07 [0.99, 1.15]	.10	0.85 [0.56, 1.26]	.43	1.16 [1.06, 1.27]	**<.001**	1.11 [0.88, 1.40]	.36

*TSA*, total shoulder arthroplasty; *ED*, emergency department; *CI*, confidence interval.

Bold indicates statistical significance with *P* ≤ .05.

∗ Multivariate analyses adjusted for surgical covariates and patient-related factors (age, sex, diabetes, Charlson comorbidity index, and smoking status).

† Parameter estimates for linear regression analysis. Reference groups include low surgical volume.

‡ Early career.

§ Non-sports medicine training.

‖ Community-based practice type.

¶ South.

In terms of fellowship training, surgeons with sports medicine and/or shoulder and elbow specialization showed lower two-year reoperation risk (OR = 0.95, CI: 0.91-0.99, *P* = .04) and fewer 90-day ED visits (OR = 0.93, 95% CI = 0.88-0.98, *P* = .002) compared to their non-sports medicine and shoulder and elbow trained counterparts. With respect to practice setting, surgeons in academic practices experienced a higher 90-day hospital readmission rate compared to community practice surgeons (OR = 1.16, 95% CI = 1.01-1.34, *P* = .03).

When compared to surgeons practicing in the South, those from the Northeast showed a lower rate of two-year reoperation (OR = 0.88, 95% CI: 0.83-0.93, *P* < .001) and revision RCR (OR = 0.88, 95% CI: 0.83-0.94, *P* < .001). Conversely, surgeons from the Midwest displayed a higher rate of conversion to TSA compared to their southern counterparts (OR = 1.62, CI: 1.25-2.10, *P* = .002). Furthermore, both Midwest and West-based surgeons exhibited higher 90-day ED visit rates compared to surgeons in the South (Midwest surgeons: OR = 1.08, 95% CI = 1.01-1.15, *P* = .01; West surgeons: OR = 1.16, 95% CI = 1.06-1.27, *P* < .001).

## Discussion

In this study, associations between surgeon-specific factors and outcomes of ARCRs were evaluated using a national administrative claims database. A novel finding was that procedural volume was associated with increased rates of reoperation and revision RCR following ARCR. Additionally, early-career surgeons had higher risks for 90-day ED visits and hospital readmissions. Surgeons who had sports medicine or shoulder and elbow fellowship training had lower rates of two-year reoperation surgery and 90-day ED visits compared to surgeons with fellowship training in other subspecialties. Additional surgeon factors such as regional practice location and practice setting demonstrated varying influences on patient outcomes.

The finding of increased revision and reoperation rates associated with higher ARCR case volumes is a novel finding as previous studies have demonstrated contrasting results of higher surgical volume being associated with lower rates of subsequent surgeries.^13^^,^^19^^,^^24^ In a systematic review and meta-analysis spanning 10 studies from 1990 to 2016, Weinheimer et al examined factors such as surgical complications, revision rates, and clinical outcomes in relation to the surgeon case volume for RCR.^29^ The findings revealed that surgeons with lower case volumes (<12 cases per year) exhibited significantly longer operation durations, extended hospital stays, and higher revision rates compared to their higher volume counterparts (>24-30 cases per year).^29^ Meanwhile, another investigation involving 518 primary ARCRs conducted by 28 surgeons did not find an association between surgeon case volume and one-year patient-reported outcome measures after primary RCR within a large hospital system.^25^ The disparities between the current study and prior reports may be attributed to several variables. Notably, the current study employed a national database, which included a much larger sample size of 94,150 procedures and accounted for surgeon-specific variables. Another plausible rationale for the current study’s unique observation is the propensity for high-volume surgeons to receive referrals for complex cases from other providers. Higher-volume surgeons might be more inclined to handle cases with greater intrinsic risks for reoperation, such as massive cuff tears, or offer revision surgeries more frequently, in contrast to their low-volume counterparts.

Early-career surgeons were found to have higher rates of 90-day hospital readmissions and ED visits than their mid-career and late-career counterparts. The elevated rates could be attributed to postoperative complications such as infections or pain management challenges.^23^^,^^30^ Additionally, early-career surgeons could be treating more medically complex patients with comorbidities, which can potentially contribute to increased rates of 90-day hospital readmissions and ED visits.^15^^,^^22^^,^^30^ Furthermore, a Medicare study comparing early-career (<3 years of practice) and experienced surgeons (at least 10 years of practice) showed that patients of early-career surgeons were generally older with more ED admissions.^16^ Lastly, surgeons in their mid-careers and late-careers may have more ancillary support, adept rehabilitation teams, and nursing personnel providing both preoperative optimization and postoperative complication monitoring.

Surgeons with training in sports medicine or shoulder and elbow fellowships demonstrated significantly lower rates of two-year rotator cuff reoperation and 90-day ED visits than their peers in other subspecialties. This may indicate that specialized expertise in shoulder surgery can lead to improved outcomes after surgery. One study that assessed the impact of fellowship training on 6-month postoperative complications after ARCR found that surgeons with sports medicine and shoulder and elbow fellowship training encountered complication rates of 11.5% and 13.5%, respectively.^15^ Comparatively, a systematic review and meta-analysis encompassing 31 studies of postoperative ARCR outcomes, without categorizing surgeon subspecialty, reported retear rates of 21% (*P* < .01) at 3-6 months and 16% (*P* < .01) at 6-12 months during the postoperative period.^18^ Collectively, these findings may suggest that the specialized expertise of sports medicine and shoulder and elbow surgeons in addressing rotator cuff pathologies might play a role in their reduced revision surgery and postoperative complication rates, a trend corroborated by our study in the context of ED visits.

Academic surgeons were found to have a higher risk of 90-day hospital readmissions compared to surgeons practicing in community settings. To our knowledge, there are no studies comparing ARCR outcomes in academic and community settings. One possible explanation for this finding is that academic surgeons generally treat patients with more medical complexities of greater severity compared to community hospitals.^5^^,^^27^ Such patients are more likely to be readmitted to the hospital after ARCR due to higher risks of complications from surgery.^10^^,^^12^^,^^21^^,^^26^ However, despite having a higher risk of 90-day hospital readmissions, academic surgeons had significantly lower rates of conversion to TSA as they may be more inclined to attempt joint-sparing procedures, such as revision RCR, superior capsular reconstruction, and tendon transfers compared to community-based surgeons.

### Limitations

Although the present study was strengthened by the large number of patients and surgeons, there were several limitations. The primary limitation was the use of a large administrative database, which is dependent on the quality and accuracy of billing codes. Miscoding and noncoding by providers may potentially omit some patients and thus misrepresent case volume or exclude certain low-volume surgeons. Moreover, to protect patient confidentiality, the database excluded surgeons performing less than 11 ARCRs during the 2015-2018 study period, thus making our results inapplicable to very low-volume surgeons. Furthermore, the dataset obtained from the Mariner database encompasses individuals insured by entities such as Medicare, Medicaid, United Healthcare, and Humana. Consequently, it is possible that some surgeons were omitted or miscategorized if their practices do not accept the insurance providers referenced in this database. Finally, we were unable to account for radiologic factors such as tear size, fatty infiltration, and surgical indications for revision due to their unavailability in the PearlDiver database.

## Conclusion

Compared to low-volume surgeons, high-volume surgeons demonstrated higher rates of total reoperation and two-year revision RCR. Early-career surgeons had elevated rates of 90-day hospitalizations and ED visits. Surgeons with fellowship training in sports medicine and shoulder and elbow surgery exhibited lower two-year reoperation rates compared to those without such specialization. Academic surgeons showed increased 90-day hospital readmission rates. These findings illustrate the impact of surgeon-specific factors on patient outcomes following ARCR.

## Disclaimers:

Funding: No funding was disclosed by the authors.

Conflicts of interest: Drew A. Lansdown reports grants from Arthrex, Inc., Smith & Nephew, and Evolution Surgical. Brian T. Feeley reports Associate editor for *Journal of Shoulder and Elbow Surgery*; research funding through NIH, consulting fees from Kaliber Labs: Consultant; grant fundings from Zimmer. C. Benjamin Ma reports grants from Anika, Samumed, and Zimmer, grants and personal fees from Histogenics, personal fees from CONMED Linvatec, Medacta, SLACK Incorporated, Wright Medical, and Stryker. Alan L. Zhang reports consulting fees from Stryker, Depuy-Mitek, Conmed, grants from Zimmer. The other authors, their immediate families, and any research foundation with which they are affiliated have not received any financial payments or other benefits from any commercial entity related to the subject of this article.
