# Supplementary material for: Impact of surgeon volume, experience, and training on outcomes after arthroscopic rotator cuff repair: a nationwide analysis of 1489 surgeons
Source: JSES Int. 2024 Apr 27;8(4):837–44. doi: 10.1016/j.jseint.2024.04.004 (PMC11258843; doi:10.1016/j.jseint.2024.04.004)
Supplement: Supplementary Table S1 [file mmc1.docx]

| Appendix Table I. CPT/ICD Codes Queried | |
| --- | --- |
| Description | CPT/ICD Codes Queried |
| Arthroscopy, shoulder, surgical; with rotator cuff repair | CPT-29827 |
|  |  |
| Evaluation and management of a patient in an emergency department | CPT-99281, CPT-99282, CPT-99283, CPT-99284, CPT-99285, CPT-G0380, CPT-G0381, CPT-G0382, CPT-G0383, CPT-G0384 |
| Shoulder-related diagnoses | ICD-9-D-72761, ICD-10-D-M75120, ICD-10-D-M75121, ICD-10-D-M75122 |
|  |  |

CPT, Current Procedural Terminology; ICD, *International Classification of Diseases*.
