# Supplementary material for: Impact of surgeon volume, experience, and training on outcomes after arthroscopic rotator cuff repair: a nationwide analysis of 1489 surgeons
Source: JSES Int. 2024 Apr 27;8(4):837–44. doi: 10.1016/j.jseint.2024.04.004 (PMC11258843; doi:10.1016/j.jseint.2024.04.004)
Supplement: Supplementary Table S2 [file mmc2.docx]

| Appendix Table II. CPT Codes Queried for Subsequent Surgeries | |
| --- | --- |
| Description | CPT/ICD Codes Queried |
| Reconstruction of complete shoulder (rotator) cuff avulsion chronic (includes acromioplasty) | CPT-23420 |
| Tenodesis of long tendon of biceps | CPT-23430 |
| Arthroplasty glenohumeral joint; total shoulder (glenoid and proximal humeral replacement (e.g., total shoulder) | CPT-23472 |
| Revision of total shoulder arthroplasty, including allograft when performed; humeral or glenoid component | CPT-23473 |
| Arthroscopy shoulder surgical; debridement limited | CPT-29822 |
| Arthroscopy shoulder surgical; debridement extensive | CPT-29823 |
| Arthroscopy shoulder surgical; distal claviculectomy including distal articular surface (Mumford procedure) | CPT-29824 |
| Arthroscopy shoulder surgical; decompression of subacromial space with partial acromioplasty with or without coracoacromial release | CPT-29826 |
| Arthroscopy shoulder surgical; with rotator cuff repair | CPT-29827 |
| Arthroscopy shoulder biceps tenodesis | CPT-29828 |
|  |  |

CPT, Current Procedural Terminology.
